# Characterization of fungal communities transmitted from sow to piglet

**DOI:** 10.1128/spectrum.02078-24

**Published:** 2025-08-25

**Authors:** Jinping Fan, Mengzhen Song, Yanguang Ling, Zhifeng Wu, Wen Xiong, Shiyu Tao, Yaoqin Shen

**Affiliations:** 1 College of Animal Sciences and Technology, Huazhong Agriculture University 627716, Wuhan, China; 2 College of Veterinary Medicine, Huazhong Agricultural University 627716, Wuhan, China; The Hebrew University-Hadassah School of Dental Medicine, Jerusalem, Israel

**Keywords:** ITS1, fungal communities, vertical transmission, sow, newborn piglets, FEAST

## Abstract

Understanding the composition of the healthy gut microbiota in neonatal piglets is critical during the early stages of life, particularly in the context of environmental change and immune development. The gut microbiota of newborn piglets is closely related to that of their sows. However, research on the characteristics of gut microbiota transmitted from mother to offspring has primarily focused on gut bacteria, with comparatively less attention given to gut fungi. In this study, vaginal and fecal samples were collected from 12 sows, along with fecal samples from 12 newborn piglets. The internal transcribed spacer 1 (ITS1) rRNA gene was sequenced to characterize the gut fungal communities. The results indicated that sow feces exhibited the greatest diversity of fungal communities, followed by piglet feces, while the least diverse fungal communities were found in sow vaginas. Significant differences in beta diversity of fungal communities were observed among the three groups, with clear segregation of the fungal communities. The composition of gut fungal communities in sow vaginas and feces, as well as in piglet feces, was dominated by Ascomycota and Basidiomycota. Fast expectation-maximization microbial source tracking (FEAST) analysis showed that 47.1 and 4.2% of the fungal communities in piglet feces originated from sow feces and sow vagina, respectively, while the remaining 48.7% were from unknown sources. At both the genus and species levels, six fungal genera or species were identified among the top fungal taxa across the three groups, suggesting that they may represent core dominant fungi transmitted from sows to piglets. Functional predictions for all three groups of fungal communities were associated with animal pathogens. These findings contribute to our understanding of the microbiological composition of the gut during early life and highlight the importance of fungal communities.

**IMPORTANCE:**

Our results highlight the association between sow fecal and piglet fecal fungal communities. The findings indicate significant differences among the fungal communities in maternal feces, maternal vagina, and piglet feces. However, the fungal communities in maternal feces and piglet feces were more similar to each other than to those found in the maternal vagina, suggesting potential vertical transmission to newborn piglets. This study describes the structure and composition of fungal communities in maternal feces, maternal vagina, and piglet feces, providing insights into the gut microbial composition during early life.

## INTRODUCTION

Early in life, gut microbes play a critical role in host health and development. The gut is home to a diverse array of microorganisms, including bacteria, fungi, archaea, protozoa, and viruses (1). While the role of gut fungi was often overlooked in past decades, several recent studies have demonstrated that fungi may significantly contribute to metabolism and gut health in both humans and mice (1–3). Although some studies have investigated fungal communities in the human and murine gut (4, 5), research on the composition and diversity of fungi in the porcine gut remains limited. Summers et al. found that *Kazachstania slooffiae* may be the predominant fungal species in weaned piglets (6), suggesting the gut fungal communities of piglets might be distinct. However, there has been relatively little research into the characterization and composition of fungal communities in neonatal piglets. The composition, characteristics, and origins of gut fungal communities in newborn piglets remain unknown.

During parturition, microorganisms are vertically transferred from the sow to the piglet, facilitating the initial colonization of the gastrointestinal tract (7, 8). Fungal colonization of the gastrointestinal tract occurs shortly after birth in both humans and pigs (9, 10) and can occur through vertical, horizontal, and environmental transmission (11, 12). Understanding the gut microbiota of lactating piglets is crucial, as it may predict the incidence and severity of post-weaning diarrhea and influence the growth performance of adult pigs (13, 14). Several studies have identified correlations between the porcine gut microbiome and animal health and production at key growth stages (15–20). Wang et al. (21) characterized the dynamics of the porcine gut microbiome by analyzing a total of 273 rectal swabs collected from birth (day 0) to market (day 174); however, changes in fungal communities were not investigated. While the effects of bacteria on immune system development have been well documented, fungi have received comparatively less attention.

Pigs serve as a valuable model for human health research due to their similarities to humans in terms of gut structure and immune system function (22). Notably, 96% of the functional pathways identified in the human gene catalog are also present in the pig gut microbiome gene catalog, underscoring the importance of pigs as a biomedical model for human studies (23). The relationship between the gut fungal communities of newborn piglets and those of their dams is currently unknown. In this study, we used ITS1 sequencing technology combined with bioinformatics analysis to analyze the characteristics and composition of fungal communities in sow feces, sow vagina, and piglet feces and compare the differences between these three groups of fungal communities. We further analyzed the origin of the fungal community in the feces of newborn piglets, as traced through the three groups of fungal operational taxonomic units (OTUs) using FEAST (24).

## RESULTS

### Sequencing data analysis

To investigate the fungal landscape of the gut in sows and neonatal piglets, we collected 12 fecal and vaginal samples from sows, as well as 12 fecal samples from neonatal piglets, for ITS sequencing. The ITS sequencing of the 36 samples generated a total of 4,133,742 raw reads, averaging approximately 114,826 reads per sample. After quality filtering, removal of chimeric sequences, and denoising, a total of 3,149,567 sequences were retained, averaging about 87,488 reads per sample, resulting in an average retention efficiency of 75% for the remaining reads in each sample. Additionally, the sequencing data, along with the total number of bases, are summarized in Table 1. Our comprehensive identification revealed a total of 7,862 OTUs, which were classified into 26 phyla, 69 classes, 151 orders, 342 families, 666 genera, and 885 species of fungi.

**TABLE 1:** Sow and piglet fungal sequencing data^*a*^

Sample	RawPE	Combined	Qualified	Nochime	Effective reads	Effective/%	Base (nt)	Avglen (nt)	GC/%	Q20/%	Q30/%
MF_2	110,280	98,352	98,028	92,381	86,791	78.7	22,743,179	246.19	51.04	99.15	96.77
MF_3	125,112	109,245	108,718	103,000	99,246	79.33	27,708,052	269.01	52.2	99.01	96.02
MF_4	130,558	118,252	117,384	113,957	68,226	52.26	36,168,797	317.39	31.88	97.6	92.32
MF_5	119,539	107,249	106,955	100,121	92,812	77.64	24,615,664	245.86	49.19	99.14	96.62
MF_6	118,417	105,345	104,920	100,261	78,789	66.54	27,686,091	276.14	38.96	98.46	94.7
MF_7	64,000	52,473	52,310	48,224	37,104	57.98	11,844,921	245.62	45.34	99.04	96.35
MF_8	109,884	96,869	96,560	90,565	87,422	79.56	22,497,139	248.41	45.67	99.24	96.78
MF_9	87,425	74,418	74,224	69,805	58,064	66.42	15,790,672	226.21	49.22	99.42	97.48
MF_10	74,690	62,233	62,132	58,603	46,428	62.16	13344059	227.7	47.81	99.35	97.31
MF_11	72,733	59,980	59,823	57,095	44,869	61.69	12,502,348	218.97	51.23	99.31	97.28
MF_12	96,370	84,321	84,004	80,308	68,974	71.57	17,754,900	221.09	50.35	99.36	97.37
MF_13	62,767	50,867	50,747	47,219	36,247	57.75	11,040,437	233.81	49.88	99.13	96.84
SZ_2	132,649	124,565	124,311	120,867	106,923	80.61	28,030,153	231.91	51.48	99.33	97.01
SZ_3	131,319	118,831	118,006	114,463	101,181	77.05	29,733,558	259.77	50.3	98.84	95.63
SZ_4	133,760	129,712	129,457	125,716	104,997	78.5	28,994,106	230.63	53.71	99.4	97.18
SZ_5	144,370	139,917	139,710	134,598	120,403	83.4	30,738,784	228.37	52.74	99.49	97.61
SZ_6	133,396	126,710	126,441	120,828	108,652	81.45	27,295,942	225.91	51.72	99.36	97.16
SZ_7	121,257	110,039	109,762	106,297	94,795	78.18	24,971,977	234.93	51.46	99.24	96.75
SZ_8	136,149	129,144	128,907	124,183	101,301	74.4	28,918,413	232.87	51.05	99.31	96.97
SZ_9	154,349	148,649	148,446	140,991	127,168	82.39	32,741,610	232.22	52.74	99.49	97.6
SZ_10	118,720	107,203	106,953	102,294	99,209	83.57	23,415,746	228.91	49.57	99.38	97.27
SZ_11	158,026	147,166	146,676	138,790	125,990	79.73	33,647,928	242.44	47.02	99.34	97.13
SZ_12	131,315	112,644	112,354	108,304	94,668	72.09	25,222,746	232.89	49.68	99.09	96.22
SZ_13	147,027	128,562	127,812	115,506	83,631	56.88	30,342,665	262.69	43.14	98.25	94.23
ZF_2	64,228	50,470	50,318	47,526	46,038	71.68	11,432,693	240.56	47.37	99.15	97.03
ZF_3	112,665	101,086	100,870	99,186	93,280	82.79	23,173,712	233.64	48.21	99.41	97.41
ZF_4	127,477	115,117	114,860	110,344	102,673	80.54	25,832,391	234.11	49.89	99.35	97.35
ZF_5	92,191	88,466	88,321	84,229	75,445	81.84	19,636,309	233.13	51.28	99.58	98.21
ZF_6	131,902	119,467	119,335	111,128	108,070	81.93	24,632,621	221.66	49.24	99.54	97.96
ZF_7	64,961	53,704	53,520	51,507	50,276	77.39	12,291,995	238.65	48.6	99.08	96.71
ZF_8	136,052	123,080	122,887	112,210	102,778	75.54	26,022,782	231.91	51.35	99.49	97.79
ZF_9	85,123	73,615	73,432	71,230	69,486	81.63	16,512,358	231.82	49.37	99.32	97.26
ZF_10	103,343	91,126	90,885	87,911	84,423	81.69	20,940,763	238.2	48.78	99.33	97.3
ZF_11	132,082	126,519	126,261	123,637	112,863	85.45	29,494,948	238.56	49.9	99.54	98.03
ZF_12	134,227	127,898	127,737	124,039	117,496	87.54	28,475,748	229.57	51.99	99.66	98.43
ZF_13	135,379	129,116	128,951	124,081	112,849	83.36	28,428,913	229.12	52.58	99.51	97.81

^*a*^ MF: maternal feces, SZ: maternal vagina, and ZF: piglet feces.

### Diversity and structure of gut fungal communities in sows and piglets

Among the fungal sequences analyzed, 76.9, 74.2, and 69% were assigned to the phylum, genus, and species levels, respectively, for fungal taxonomy. The increasing number of unclassified genera and species identified in the fungal sequences has resulted in a decreasing rate of sequence assignment from phylum to species. The small differences in the proportions of phyla, genera, and species may be attributed to the higher resolution of fungal sequence identifications.

**Fig 1:**
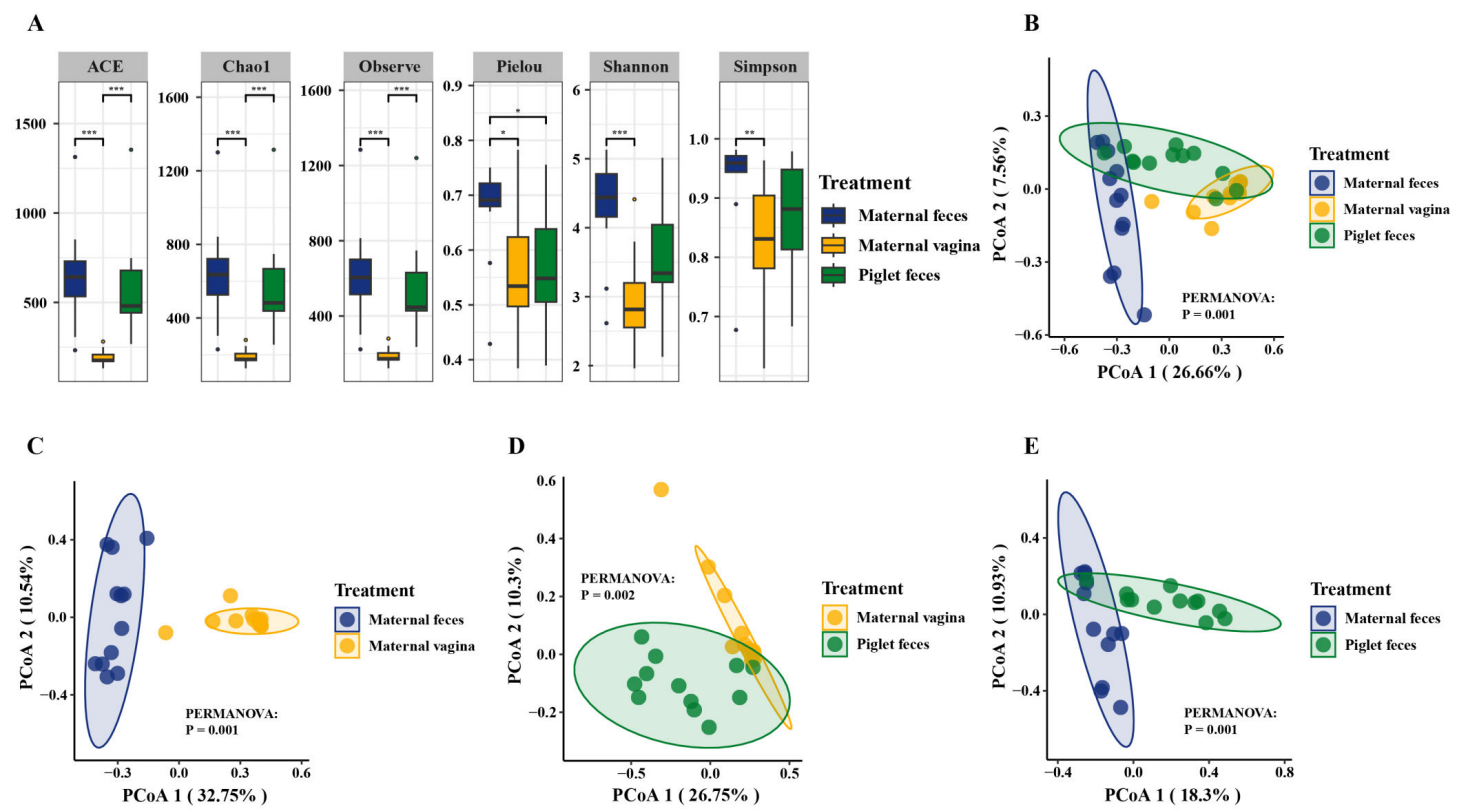
Diversity and structure of gut fungal communities in sows and piglets. (**A**) Comparison of the alpha diversities of gut fungal communities between sows and piglets. (**B**) PCoA analysis of gut fungal communities between sows and piglets. PCoA analysis of gut fungal communities between maternal feces and maternal vagina (**C**), maternal vagina and piglet feces (**D**), and maternal feces and piglet feces (**E**). *P < 0.05, **P < 0.01, and ***P < 0.001. *n* = 12.

In this study, the Chao1 and ACE indices were utilized to evaluate fungal richness, while the Shannon and Simpson indices were applied to assess fungal diversity. The alpha diversity analysis (Fig. 1A) indicated that the Chao1 and ACE indices for the fungal communities were significantly lower in the maternal vagina group compared to the maternal feces and piglet feces groups (*P* < 0.001). Furthermore, the Shannon and Simpson indices of the fungal communities were significantly higher in the maternal feces group than the maternal vagina group (*P* < 0.01). Based on the Chao1, ACE, Shannon, and Simpson indices, the alpha diversity of the fungal communities in the maternal feces and piglet feces groups was significantly greater than that in the maternal vagina group.

Beta diversity analysis, as indicated by the principal coordinate analysis (PCoA) results (PERMANOVA; *P* = 0.001; Fig. 1B), demonstrated that the fungal community structures among the three groups were significantly distinct from one another. Additionally, pairwise comparisons revealed clear separations in the fungal community structures between the maternal feces and maternal vagina (PERMANOVA; *P* = 0.001; Fig. 1C), the maternal vagina and piglet feces (PERMANOVA; *P* = 0.002; Fig. 1D), and the maternal feces and piglet feces (PERMANOVA; *P* = 0.001; Fig. 1E).

### Composition of gut fungal communities in sows and piglets

**Fig 2:**
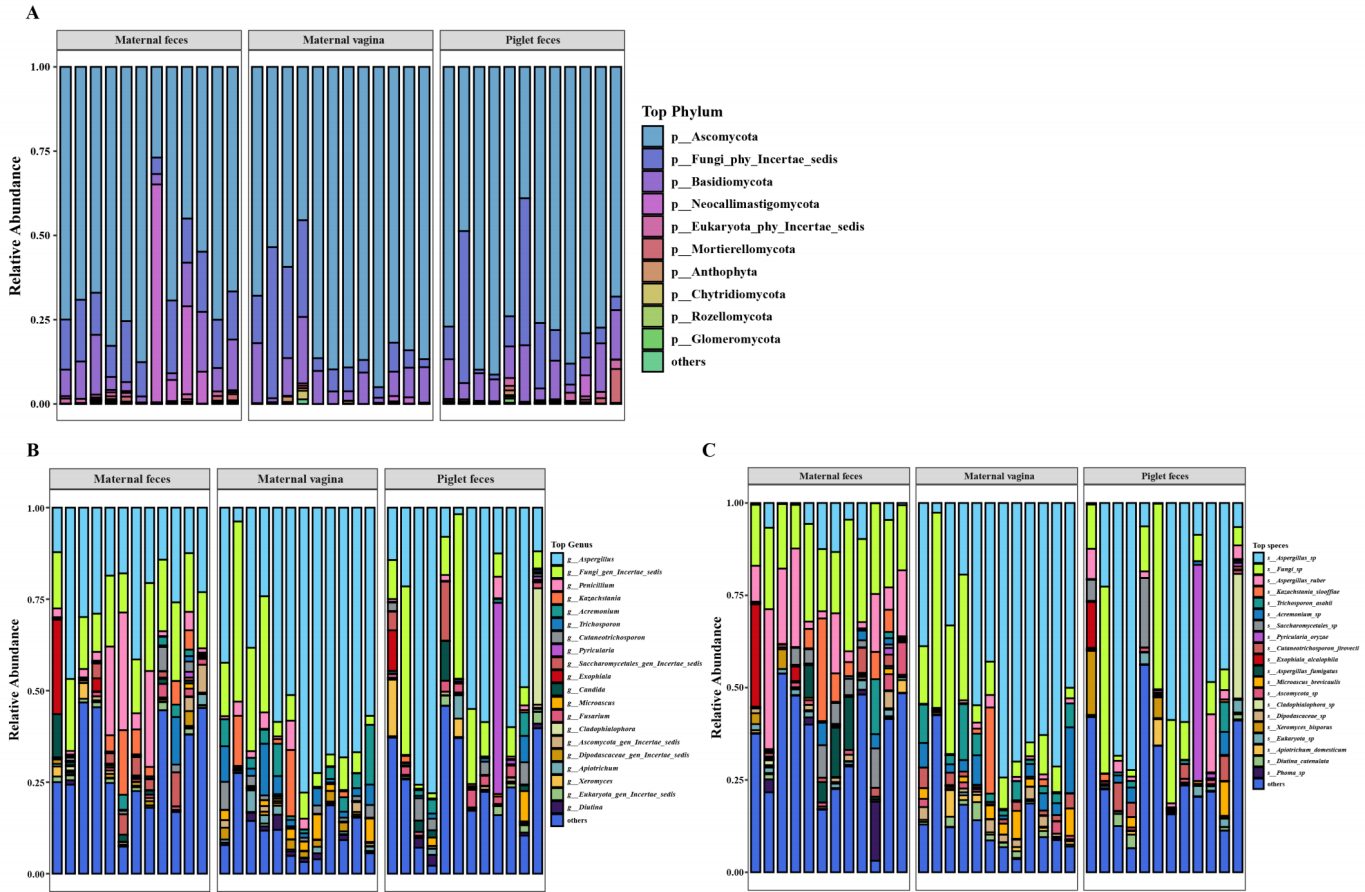
Composition of gut fungal communities in sows and piglets at the phylum (**A**), genus (**B**), and species (**C**) levels.

At the phylum level (top 10 relative abundance), the fungi found in maternal feces were dominated by Ascomycota, Fungi_phy_Incertae_sedis, Neocallimastigomycota, and Basidiomycota, which accounted for 66.2, 14.1, 9.1, and 8.6% of the total fungal phyla, respectively (Fig. 2A). The fungi in the maternal vagina were primarily composed of Ascomycota, Fungi_phy_Incertae_sedis, and Basidiomycota, accounting for 77.2, 12.9, and 8.6% of the total fungal phyla, respectively (Fig. 2A). The fungi in piglet feces were dominated by Ascomycota, Fungi_phy_Incertae_sedis, and Basidiomycota comprising 73.9, 13.4, and 9.1% of the total fungal phyla, respectively (Fig. 2A). Ascomycota was the most dominant phylum among the fungi, showing a significantly higher abundance than other phyla, accounting for 66.2 to 77.2% of all fungal phyla obtained from the three groups. Additionally, the relative abundance of Neocallimastigomycota was significantly lower in the maternal vagina group compared to the maternal feces and piglet feces groups (*P* < 0.05).

At the genus level, the fungi in maternal feces were dominated by *Aspergillus*, *Fungi_gen_Incertae_sedis*, *Penicillium*, and *Kazachstania*, which accounted for 24.3, 16.7, 8.6, and 3.6% of the total fungal genera, respectively (Fig. 2B). The fungi in the maternal vagina were primarily composed of *Aspergillus*, *Fungi_gen_Incertae_sedis*, *Acremonium*, and *Trichosporon*, accounting for 52.3, 13.8, 4.3, and 4.1% of the total fungal genera, respectively (Fig. 2B). The fungi in piglet feces were mainly dominated by *Aspergillus*, *Fungi_gen_Incertae_sedis*, and *Pyricularia* comprising 37.2, 14.0, and 4.6% of the total fungal genera, respectively (Fig. 2B). *Aspergillus* was the most dominant genus among the fungi, exhibiting a significantly higher abundance than other genera and accounting for 24.3 to 52.3% of all fungal genera obtained from the three groups. Furthermore, the relative abundance of *Acremonium* and *Trichosporon* was significantly higher in the maternal vagina group compared to the maternal feces and piglet feces groups (*P* < 0.05).

At the species level, the fungi in maternal feces were dominated by *Fungi_sp*, *Aspergillus_ruber*, and *Aspergillus_sp*, accounting for 20.5, 13.3, and 3.6% of the total fungal species, respectively (Fig. 2C). The fungi in the maternal vagina were primarily composed of *Aspergillus_sp*, *Fungi_sp*, and *Trichosporon_asahii*, accounting for 48.8, 15.7, and 4.6% of the total fungal species, respectively (Fig. 2C). The fungi in piglet feces were mainly dominated by *Aspergillus_sp*, *Fungi_sp*, and *Pyricularia_oryzae*, comprising 33.1, 15.6, and 5.1% of the total fungal species, respectively (Fig. 2C). Additionally, the abundance of *Aspergillus_ruber* and *Fungi_sp* in the maternal feces group was significantly higher than in the maternal vagina and piglet feces groups, whereas *Aspergillus_sp* was significantly lower (*P* < 0.05).

### Source tracking of fungal microbiota

**Fig 3:**
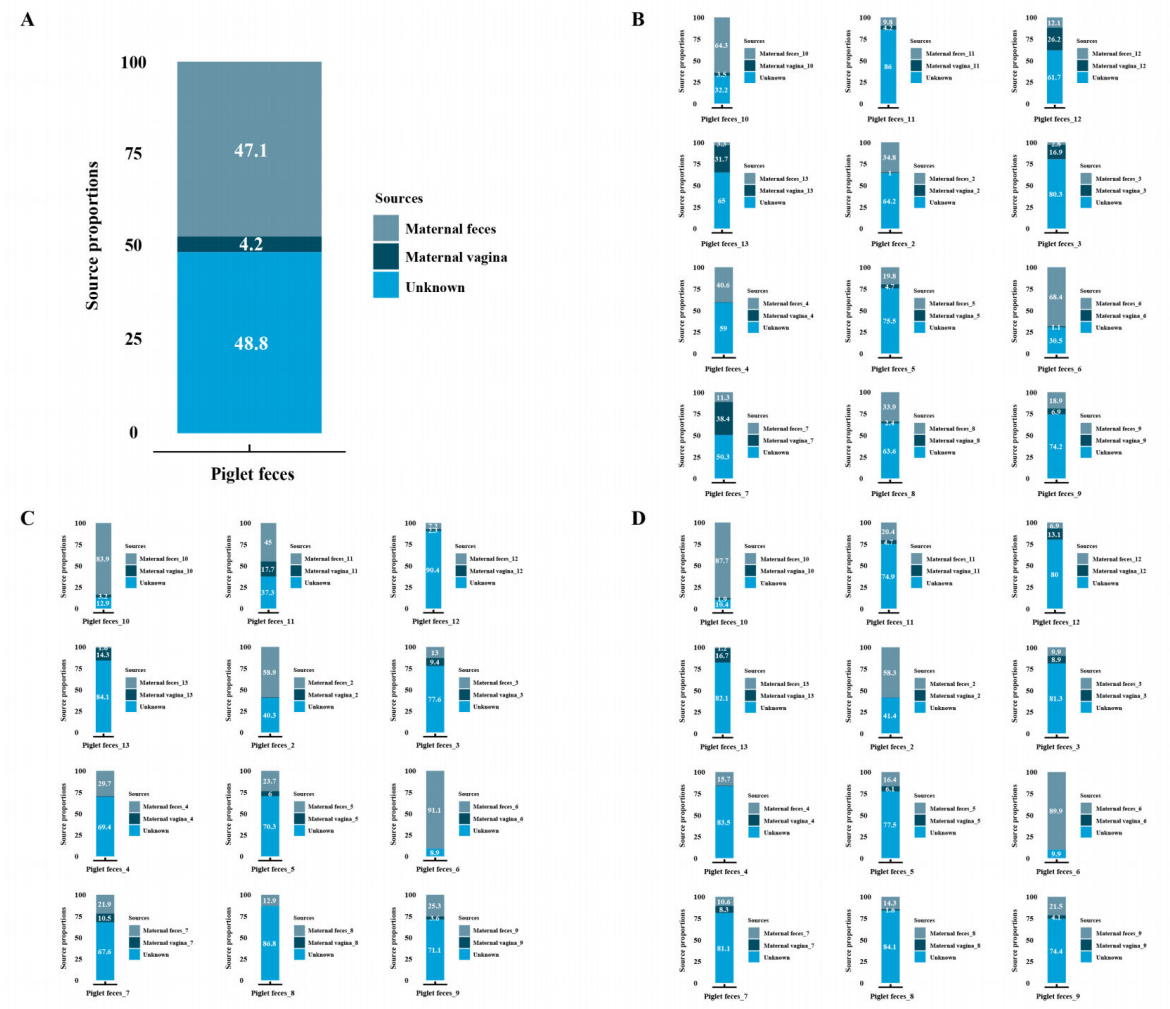
Source tracking of fungal microbiota. Tracking the source of the gut fungal communities in average (**A**) and individual piglets (**B**) at the OTU level. Tracking the source of the gut fungal communities in individual piglets at the genus (**C**) and species (**D**) levels.

Notably, despite the differences in the structure of the fungal communities, the species composition of the fungal communities in the three groups was similar. Based on the common fungal communities found in maternal feces, maternal vagina, and piglet feces, we hypothesized that the fungal communities in piglet feces may originate from sow feces or sow vaginas. The FEAST was employed to test this hypothesis and identify the source of the fungal communities in piglet feces. On average, 47.1% of the OTUs in piglet feces were estimated to originate from maternal feces, and 4.2% of the OTUs in piglet feces were estimated to originate from the maternal vagina (Fig. 3A). However, approximately 48.7% of the OTUs in piglet feces were also predicted to be from unknown sources (i.e., sites not sampled or considered in this analysis). The results of tracking the sources of the fungal communities in the feces of individual piglets confirmed that the primary source of fungi in piglet feces was maternal feces (Fig. 3B). Interestingly, we found similar results when tracking the sources of the fungal communities at the genus (Fig. 3C) and species (Fig. 3D) levels. Thus, these results indicate that the fungal communities in the piglet gut mainly derive from maternal intestinal sources, with a small proportion coming from the maternal vagina.

### Differential analysis of gut fungal communities and core-predominant gut fungi in sows and piglets

**Fig 4:**
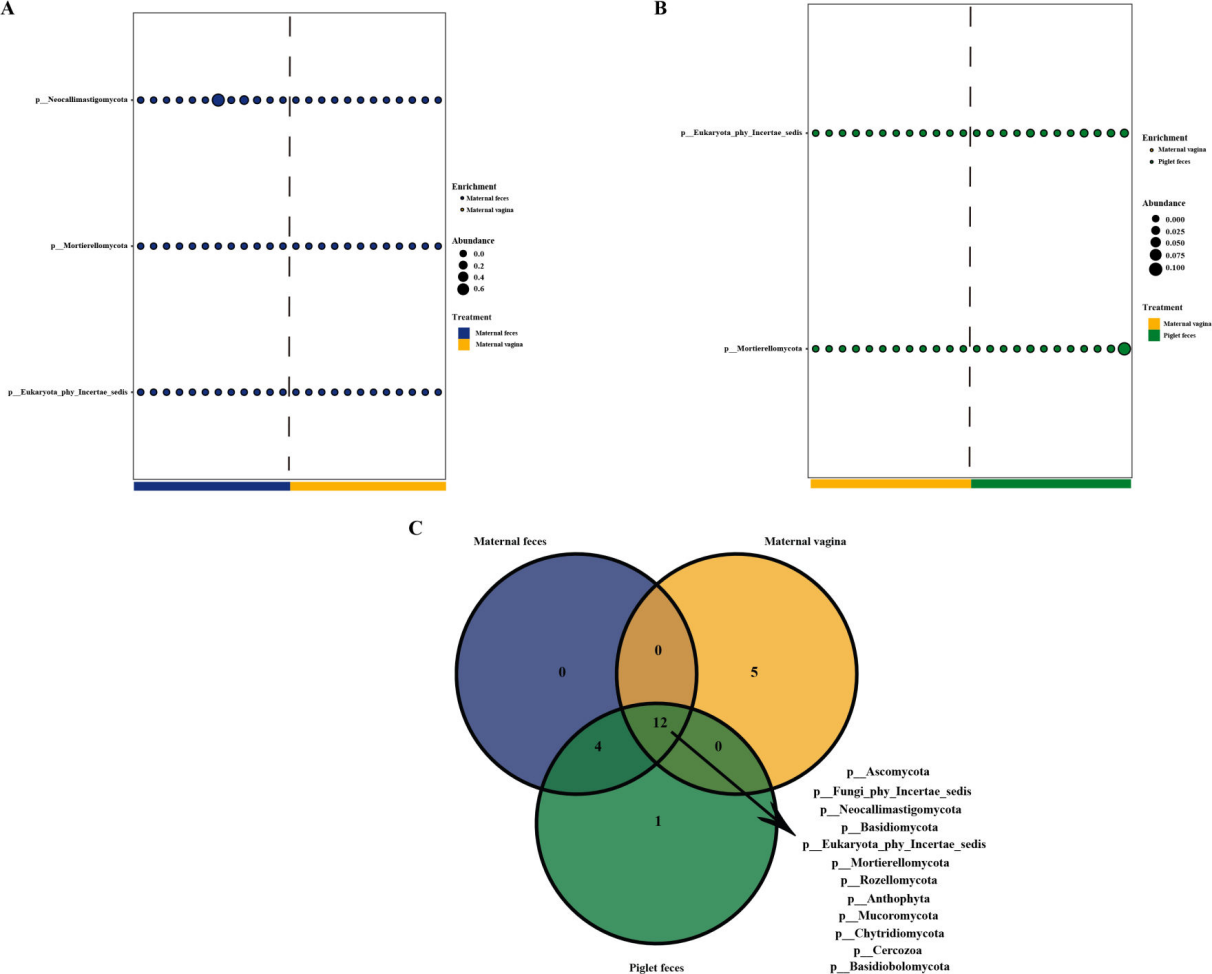
Differential analysis of gut fungal communities in sows and piglets. Intestinal differential fungal between maternal feces and maternal vagina groups (**A**) and maternal vagina and piglet feces groups (**B**) at phylum level. (**C**) Core-predominant fungal phyla of all fungal phyla in the three groups. *n* = 12.

We analyzed differences in the gut fungal communities of pigs and identified core predominant gut fungi. At the phylum level, compared to the maternal vagina group, the maternal feces and piglet feces groups exhibited three and two fungal phyla with higher relative abundance, respectively (*P* < 0.05; Fig. 4A and B). Differential analysis showed no statistically significant difference in the relative abundance of fungal phyla between the maternal feces and piglet feces groups. Furthermore, the Venn diagram (Fig. 4C) indicated that 12 core-predominant fungal phyla (including Ascomycota, Fungi_phy_Incertae_sedis, Neocallimastigomycota, Basidiomycota, Eukaryota_phy_Incertae_sedis, Mortierellomycota, Rozellomycota, Anthophyta, Mucoromycota, Chytridiomycota, Cercozoa, and Basidiobolomycota) were shared among the fungal phyla in the three groups.

**Fig 5:**
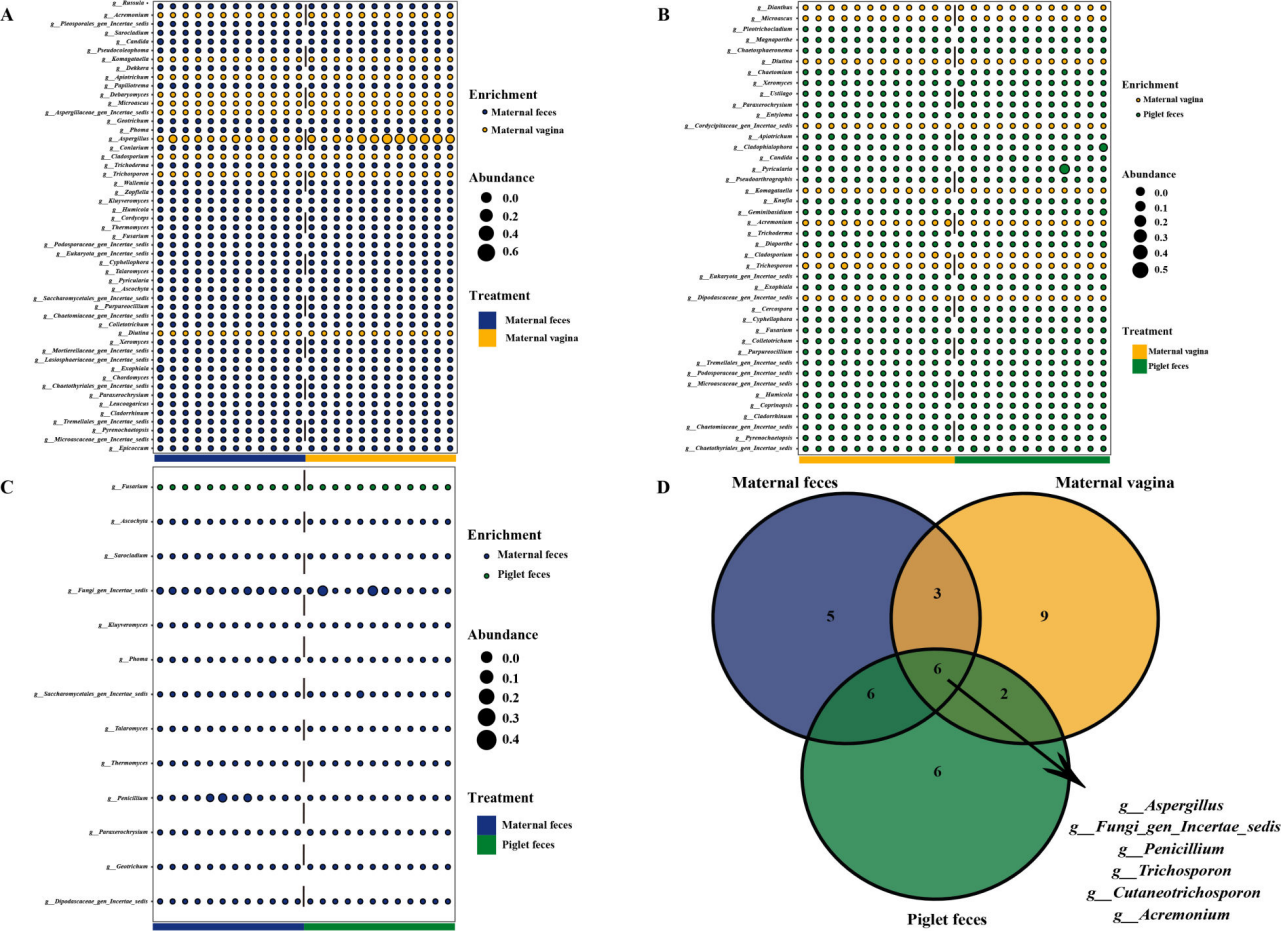
Differential analysis of gut fungal communities in sows and piglets. Intestinal differential fungal communities between maternal feces and maternal vagina groups (**A**), maternal vagina and piglet feces groups (**B**), maternal feces and piglet feces groups (**C**) at genus level. (**D**) Core-predominant fungal genera of the top 20 relative abundant fungal genera in the three groups. *n* = 12.

At the genus level, compared to the maternal vagina group, the maternal feces and piglet feces groups contained 41 and 33 fungal genera of high relative abundance and 10 and nine fungal genera of low relative abundance, respectively (*P* < 0.05; Fig. 5A and B). In comparison to the maternal feces group, the piglet feces group exhibited 12 fungal genera of high relative abundance and one fungal genus of low relative abundance (*P* < 0.05; Fig. 5C). The Venn diagram (Fig. 5D) further demonstrated that six core-predominant fungal genera (including *Aspergillus*, *Fungi_gen_Incertae_sedis*, *Penicillium*, *Trichosporon*, *Cutaneotrichosporon*, and *Acremonium*) were shared among the top 20 most abundant fungal genera in the three groups.

**Fig 6:**
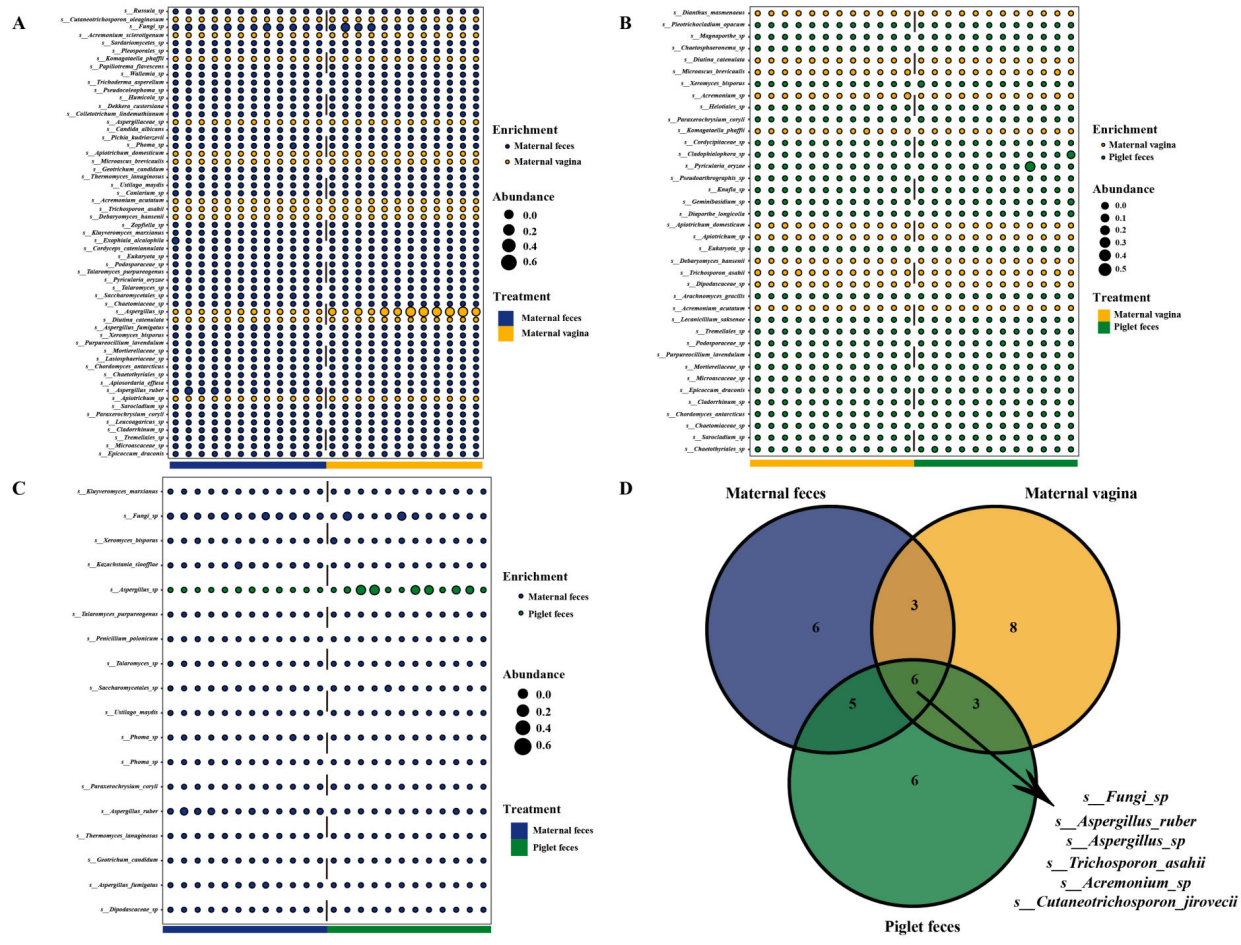
Differential analysis of gut fungal communities in sows and piglets. Intestinal differential fungal between maternal feces and maternal vagina groups (**A**), maternal vagina and piglet feces groups (**B**), and maternal feces and piglet feces groups (**C**) at species level. (**D**) Core-predominant fungal species of the top 20 relative abundant fungal species in the three groups. *n* = 12.

At the species level, compared to the maternal vagina group, the maternal feces and piglet feces groups displayed 45 and 27 fungal species of high relative abundance and 12 and 11 fungal species of low relative abundance, respectively (*P* < 0.05; Fig. 6A and B). Compared to the maternal feces group, the piglet feces group had 17 fungal species of high relative abundance and one fungal species of low relative abundance (*P* < 0.05; Fig. 6C). Additionally, the Venn diagram (Fig. 6D) revealed that six core-predominant fungal species (including *Fungi_sp*, *Aspergillus_ruber*, *Aspergillus_sp*, *Trichosporon_asahii*, *Acremonium_sp*, and *Cutaneotrichosporon_jirovecii*) were shared among the top 20 most abundant fungal species in the three groups.

### Functional prediction of gut fungal communities in sows and piglets

**Fig 7:**
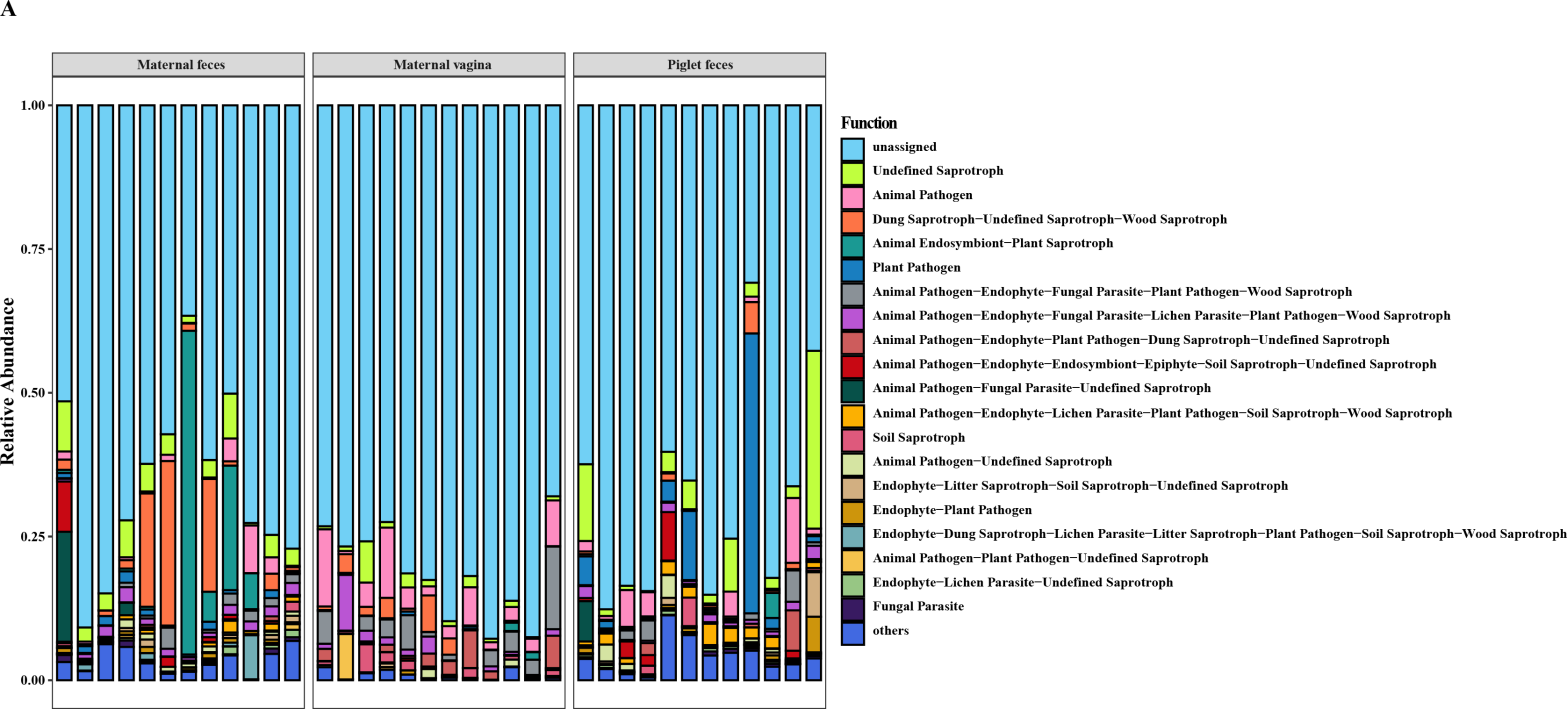
Functional prediction of gut fungal communities in sows and piglets. (**A**) Functional prediction of three groups of intestinal fungal communities using FUNGuild. *n* = 12.

PICRUSt predictions do not apply to fungal OTUs and therefore cannot directly predict fungal function. However, by utilizing the FUNGuild software, which describes fungal nutritional patterns, we were able to gain a general understanding of the gut fungal function in sows and piglets. We further predicted the functions of the fungal communities in the three groups. FUNGuild classified these into three main trophic modes: pathotroph, saprotroph, and symbiotroph. This study primarily focused on Pathotroph and Saprotroph modes. The histogram of fungal functions (Fig. 7A) indicated that most functions were unknown, while the known functions were mainly associated with saprotroph and animal pathogen categories.

In maternal feces, known fungal functions were primarily classified as dung saprotroph-undefined saprotroph-wood saprotroph and animal endosymbiont-plant saprotroph. In contrast, the known fungal functions in the maternal vagina were predominantly animal pathogen. However, the functions of gut fungi in piglets varied due to individual differences among sows.

## DISCUSSION

Mother-to-child transmission of microorganisms, particularly viruses (25) and bacteria (26), has been extensively studied, while the transmission of fungi has received less attention. In this study, we characterized the intestinal fungal communities of sows and newborn piglets from the perspective of mother-to-child transmission. We analyzed their diversity, composition, traceability, variation, core-predominant fungi, and functional predictions.

Our findings revealed significant differences in fungal communities between the gut and vagina of sows, as well as in the gut of newborn piglets. The main fungal phyla identified in the three groups were Ascomycota, Fungi_phy_incertae_sedis, and Basidiomycota. The traceability analysis of intestinal fungi in newborn piglets suggested that their gut fungi are more likely to originate from the intestines of sows rather than from the maternal vagina. Notably, the differences in gut fungal communities between newborn piglets and sows were smaller compared to those observed between the sow’s gut and vagina. Additionally, we analyzed the core fungi shared among the three groups. A total of 3,149,567 fungal sequences were obtained from the maternal vaginal and fecal samples, as well as piglet fecal samples, resulting in the identification of 7,862 OTUs. This was significantly higher than the 2,304,968 fungal sequences generated from fecal samples of three different pig breeds, which yielded 6,942 clustered OTUs (27). We identified 26 phyla and 666 genera, far exceeding the six phyla and 148 genera identified by Li et al. (27). However, many fungi remain unidentified, indicating that the intestinal fungal communities of sows and neonatal piglets warrant further exploration.

The high coverage, Q20, and Q30 values (Table 1) indicate a high quality of the fungal sequences obtained in this study. Based on the Chao1, ACE, Shannon, and Simpson indices, we observed the greatest diversity of fungal communities in maternal feces, followed by piglet feces, with the least diversity found in maternal vagina samples. This finding is consistent with previous results on bacterial diversity in human maternal and infant feces, where the Shannon diversity of maternal fecal bacteria was significantly higher than that of infant feces (28).

Moreover, the beta diversity of the fungal communities across the three groups was significantly different. We noted a clear separation in the fungal community structure between the maternal feces and maternal vagina groups. However, there was overlap between the piglet feces, maternal feces, and maternal vagina groups, suggesting that the fungi in piglet feces may have originated from both sources.

In our study, the gut fungal communities in the maternal vagina and feces, as well as in piglet feces, were predominantly composed of Ascomycota and Basidiomycota, consistent with previous studies in humans and mice (2, 29, 30), weaned piglets (31), cattle (32), and sheep (33). Additionally, we found that the third most abundant fungal phylum in maternal feces was Neocallimastigomycota. Interestingly, the second most abundant fungal phylum across the three groups was Fungi_phy_Incertae_sedis. Overall, the dominant fungal genus in the three groups was primarily *Aspergillus*. Furthermore, the three groups of fungal communities exhibited differences in the top three relative abundances at both the phylum and genus levels.

Davis et al. (28) found that maternal fecal bacterial diversity was highest at all sites sampled from both mothers and infants, with maternal feces and breast milk identified as the primary sources of fecal microorganisms for infants. Our traceability analyses using FEAST indicated that a greater proportion of the piglet fecal fungal communities originated from maternal feces, with some also deriving from maternal vagina. Given the known sources of fungal communities in individual piglet feces in our study, we would expect a relatively significant contribution from maternal feces. Variability in the fungal communities of maternal feces may partly account for this substantial contribution.

While Davis et al. (28) focused on the mother-to-child transmission of bacteria, our findings parallel theirs, though we did not assess the role of breast milk in this context. Fecal samples were collected from newborn piglets immediately after farrowing, and since the exposure of piglets to the environment was inevitable, we cannot exclude the possibility of colonization by unknown fungi from the postnatal environment (28). Prisnee et al. (34) demonstrated that antibiotic treatment altered the bacterial composition but not the fungal composition, suggesting that maternal fungal communities play a critical role in stabilizing early piglet fungal communities. Thus, based on existing research, it is more likely that the fecal fungal communities in neonatal piglets are vertically transmitted from the sow rather than being rapidly colonized by fungi from other sources.

Hu et al. (35) identified *K. sloofiae* as the most abundant fungal species in the intestinal tract of weaned piglets. In our analysis, however, *K. sloofiae* was found to be more prevalent in sow feces and vagina, while it was almost absent in piglet feces. Zhou et al. (36) reported that *Candida tropicalis* was the most abundant species in piglet feces; however, our results showed that all three groups had almost no *C. tropicalis*. These discrepancies may be attributed to differences in pig breeds, developmental stages, and the fungal sequencing methods employed.

Differential analysis revealed that the fungi differing between maternal feces and maternal vagina, as well as between maternal vagina and piglet feces, were significantly more abundant than those differing between maternal feces and piglet feces. In contrast, maternal feces compared to piglet feces exhibited minimal differences at the phylum, genus, and species levels.

Consistent with the substantial differences observed among the three groups of fungal communities, we found that their predictive functions varied significantly. The high proportion of unknown functions suggests that our understanding of fungal functions is still limited. The substantial percentage of “Undefined Saprotrophs” among the fungal taxa may contribute to the breakdown of indigestible dietary fiber and the redistribution of nutrients (37). In soil, secondary metabolites produced by saprophytic fungi play a crucial role in the initial degradation of complex organic compounds (38).

Although all sows and piglets appeared healthy, many intestinal fungi were classified within pathogenic guilds. Notably, the known fungal functions in the maternal vagina predominantly involve animal pathogens. For example, *Aspergillus* is the fungal genus with the highest abundance in sow feces and vagina and piglet feces. Aspergillosis is less common in pigs but has been implicated as a cause of abortion in pigs (39–42). Previous studies have shown that *Aspergillus* can directly interact with bacterial species, such as *Stenotrophomonas* and *Pseudomonas*, and exacerbate disease severity (43–45). Maternal excretion of pathogenic fungi through fecal and vaginal excretion to maintain the stability of their own fungal communities may be beneficial in maintaining health (46, 47). Plant pathogenic and ectomycorrhizal fungi naturally exist in plants and soil (48, 49), and their presence in the pig intestine may result from dietary intake or soil contact. Unlike humans and model animals, pigs have greater opportunities to interact with soil, and their behavior of digging with their noses may also facilitate the introduction of soil fungi into their intestines (50). However, the gut fungal functions of newborn piglets did not exhibit significant consistency. It is noteworthy that the gut fungal functions of newborn piglets are related to the gut and vaginal fungi of their sows, which aligns with the traceability results mentioned earlier. Even low levels of pathogenic fungi transferred from the sow’s vagina can benefit piglet health by inducing innate immune responses (e.g., NETs) that enhance host defense and stimulate the maturation of the piglet’s immune system (36).

In conclusion, we have mapped the characteristic spectrum of fungal communities in sow feces, sow vagina, and piglet feces and revealed the transmission characteristics of fungi from the sow gut (but not the sow vagina) to the piglet gut. These findings deepen our understanding of the characteristics of sow-piglet fungal transmission and provide a basis for subsequent analyses of interactions between core fungi and their hosts.

## MATERIALS AND METHODS

### Animals and sample collection

Sows (Landrace × Yorkshire) and newborn piglets [Duroc × (Landrace × Yorkshire), DLY] were housed at the Tianpeng Herd in Langfang, Hebei Province, China. The herd consisted of 12 sows awaiting parturition, and fecal samples were collected from the sows after parturition, and vaginal microbiological samples were collected from 12 sows using swabs. Fecal samples were collected from one newborn piglet from each of 12 sows after birth. All specimens were divided into two portions: one portion was stored in liquid nitrogen for internal transcribed spacer 1 (ITS1) rRNA sequencing, while the other was placed in PBS with 20% glycerol.

### DNA extraction and high-throughput sequencing

Total genomic DNA was extracted from the samples using the CTAB method. DNA concentration and purity were assessed using 1% agarose gels. Based on the concentration, DNA was diluted to 1 ng/µL using sterile water. Internal transcribed spacer (ITS) identification refers to the DNA sequencing of ITS sequences, and by comparing the sequenced ITS sequences with known fungal ITS sequences, it is a method of obtaining information about the genus of the fungus being tested (51). The fungal internal transcribed spacer 1 (ITS1) region of the ribosomal RNA gene was selected for taxonomic analysis in this study due to its higher sequence variability compared to the ITS2 region. The ITS1 region is more widely used for studying fungal diversity, and a greater number of sequences for this region can be found in databases.

We amplified the ITS1 region using the primer pairs ITS1-1F (52) (5′-CTTGGTCATTTAGAGGAAGTAA-3′, 5′-GCTGCGTTCTTCATCGATGC-3′). All PCR reactions were performed with 15 µL of Phusion High-fidelity PCR Master Mix (New England Biolabs), 2 µM of forward and reverse primers, and approximately 10 ng of template DNA. Thermal cycling conditions included an initial denaturation step at 98°C for 1 min, followed by 30 cycles of denaturation at 98°C for 10 s, annealing at 50°C for 30 s, and elongation at 72°C for 30 s, concluding with a final elongation at 72°C for 5 min.

An equal volume of 1× loading buffer (containing SYBR Green) was mixed with the PCR products, and electrophoresis was performed on a 2% agarose gel for detection. The PCR products were combined in equimolar ratios, and then purified using the Qiagen Gel Extraction Kit (Qiagen, Germany).

Sequencing libraries were generated using the TruSeq DNA PCR-free Sample Preparation Kit (Illumina, USA) following the manufacturer’s recommendations, and index codes were added. The quality of the library was assessed using a Qubit 2.0 Fluorometer (Thermo Scientific) and an Agilent Bioanalyzer 2100 System. Finally, the library was sequenced on an Illumina NovaSeq platform, generating 250 bp paired-end reads.

### Sequence processing

Sequencing analyses were performed using Quantitative Insight into Microbial Ecology 2 (QIIME 2) (v2021.4) (53). Demultiplexing, quality filtering, denoising, and chimera filtering were conducted using the Split Amplicon Denoising Algorithm version 2 plug-in (54). Amplicon sequence variants (ASVs) were aligned using MAFFT (55). The resulting ASVs were classified using the classify-sklearn naive Bayes classifier (via the q2-feature-class plug-in with reference to the UNITE database version 8.3) (56).

### Diversity analysis

Following processing in QIIME 2, the biom-format data were converted into a text file for the analysis of the fungal community diversity. The R package phyloseq (v1.34.0) was employed to analyze the structure and diversity of microbial communities (57). Alpha diversity was measured using the Shannon index. Changes in overall fungal community composition were assessed using Bray-Curtis dissimilarity and permutational multivariate analysis of variance (PERMANOVA) and visualized using principal coordinate analysis (PCoA) (R v4.0.5). The analysis utilized the “GUnifrac,” “ggplot2,” and “vegan” packages within RStudio (58, 59). The Bray-Curtis index, which reflects species relative abundances without considering evolutionary relationships, served as the basis for measuring similarities among fungal communities. Interspecific comparisons were conducted using the “vegan” and “ggplot2” packages in RStudio (58). Additional visualization and analysis of fungal diversity were performed using the “pheatmap,” “ggtern,” “ggpubr,” and “circlize” packages in Rstudio (60–62).

Fast expectation-maximization microbial source tracking (FEAST), a Bayesian classifier method, was utilized to identify the sources of bacterial communities following guidelines published at https://github.com/cozygene/FEAST (24).

### Taxonomic and functional classification

Utilizing the q2-feature-classifier plugin, the classifier was calibrated with the UNITE QIIME for Fungi Version 16.10.2022 Database, which is specifically designed for fungal taxonomy and contains over 1 million annotated, quality-controlled fungal sequences (63). The Naive Bayes method was employed for sequence classification, and sequences that could not be assigned to the genus level using this method were further analyzed using UNITE online BLAST and GenBank BLAST for more precise identification. In a Python 3 environment, the FUNGuild script was used to predict fungal functional groups (64). This tool categorizes fungi into various functional groups based on criteria, such as ecological niche, nutritional mode, and host range, including lignin decomposers, pathogens, endophytes, and others.

### Statistical analysis

Alpha diversity analyses, including richness, Shannon, and Simpson index determination and visualization, were performed in R using the tidyverse (version 1.3.1), vegan (version 2.5–7), and ade4 (version 1.7–17) packages, respectively. Overall differences and similarities in fungal communities were assessed visually using PCoA, and statistical significance between groups was calculated using permutational multivariate analysis of variance (PERMANOVA). Fungal species that differed between the two groups were identified using the Wilcoxon rank sum test, and a *P* value < 0.05 was considered statistically significant.

## Data Availability

The ITS-rDNA sequences corresponding to all the samples have been submitted to the NCBI Sequence Read Archive under the accession number PRJNA1141162.

## References

[1] Qin J, Li R, Raes J, Arumugam M, Burgdorf KS, Manichanh C, Nielsen T, Pons N, Levenez F, Yamada T, et al.. 2010. A human gut microbial gene catalogue established by metagenomic sequencing. Nature 464:59–65. doi:10.1038/nature08821 20203603 PMC3779803

[2] Hoffmann C, Dollive S, Grunberg S, Chen J, Li H, Wu GD, Lewis JD, Bushman FD. 2013. Archaea and fungi of the human gut microbiome: correlations with diet and bacterial residents. PLoS One 8:e66019. doi:10.1371/journal.pone.0066019 23799070 PMC3684604

[3] Heisel T, Montassier E, Johnson A, Al-Ghalith G, Lin YW, Wei LN, Knights D, Gale CA. 2017. High-fat diet changes fungal microbiomes and interkingdom relationships in the murine gut. mSphere 2:e00351-17. doi:10.1128/mSphere.00351-17 29034327 PMC5636226

[4] Suhr MJ, Hallen-Adams HE. 2015. The human gut mycobiome: pitfalls and potentials—a mycologist’s perspective. Mycologia 107:1057–1073. doi:10.3852/15-147 26354806

[5] Li J, Chen D, Yu B, He J, Zheng P, Mao X, Yu J, Luo J, Tian G, Huang Z, Luo Y. 2018. Fungi in gastrointestinal tracts of human and mice: from community to functions. Microb Ecol 75:821–829. doi:10.1007/s00248-017-1105-9 29110065

[6] Summers KL, Frey JF, Ramsay TG, Arfken AM. 2019. The piglet mycobiome during the weaning transition: a pilot study. J Anim Sci 97:2889–2900. doi:10.1093/jas/skz182 31136650 PMC6606507

[7] Jašarević E, Howerton CL, Howard CD, Bale TL. 2015. Alterations in the vaginal microbiome by maternal stress are associated with metabolic reprogramming of the offspring gut and brain. Endocrinology 156:3265–3276. doi:10.1210/en.2015-1177 26079804 PMC4541625

[8] Aviles-Rosa EO, Rakhshandeh A, McGlone JJ. 2019. Preliminary study: depriving piglets of maternal feces for the first seven days post-partum changes piglet physiology and performance before and after weaning. Animals (Basel) 9:268. doi:10.3390/ani9050268 31126021 PMC6562806

[9] Palmer C, Bik EM, DiGiulio DB, Relman DA, Brown PO. 2007. Development of the human infant intestinal microbiota. PLoS Biol 5:e177. doi:10.1371/journal.pbio.0050177 17594176 PMC1896187

[10] Arfken AM, Frey JF, Summers KL. 2020. Temporal dynamics of the gut bacteriome and mycobiome in the weanling pig. Microorganisms 8:868. doi:10.3390/microorganisms8060868 32526857 PMC7356342

[11] Bliss JM, Basavegowda KP, Watson WJ, Sheikh AU, Ryan RM. 2008. Vertical and horizontal transmission of Candida albicans in very low birth weight infants using DNA fingerprinting techniques. Pediatr Infect Dis J 27:231–235. doi:10.1097/INF.0b013e31815bb69d 18277930

[12] Ward TL, Dominguez-Bello MG, Heisel T, Al-Ghalith G, Knights D, Gale CA. 2018. Development of the human mycobiome over the first month of life and across body sites. mSystems 3:e00140-17. doi:10.1128/mSystems.00140-17 29546248 PMC5840654

[13] Dou S, Gadonna-Widehem P, Rome V, Hamoudi D, Rhazi L, Lakhal L, Larcher T, Bahi-Jaber N, Pinon-Quintana A, Guyonvarch A, Huërou-Luron ILE, Abdennebi-Najar L. 2017. Characterisation of early-life fecal microbiota in susceptible and healthy pigs to post-weaning diarrhoea. PLoS One 12:e0169851. doi:10.1371/journal.pone.0169851 28072880 PMC5225014

[14] Maltecca C, Lu D, Schillebeeckx C, McNulty NP, Schwab C, Shull C, Tiezzi F. 2019. Predicting growth and carcass traits in swine using microbiome data and machine learning algorithms. Sci Rep 9:6574. doi:10.1038/s41598-019-43031-x 31024050 PMC6484031

[15] Ma L, Tao S, Song T, Lyu W, Li Y, Wang W, Shen Q, Ni Y, Zhu J, Zhao J, Yang H, Xiao Y. 2024. Clostridium butyricum and carbohydrate active enzymes contribute to the reduced fat deposition in pigs. Imeta 3:e160. doi:10.1002/imt2.160 38868506 PMC10989082

[16] Ramayo-Caldas Y, Mach N, Lepage P, Levenez F, Denis C, Lemonnier G, Leplat JJ, Billon Y, Berri M, Doré J, Rogel-Gaillard C, Estellé J. 2016. Phylogenetic network analysis applied to pig gut microbiota identifies an ecosystem structure linked with growth traits. ISME J 10:2973–2977. doi:10.1038/ismej.2016.77 27177190 PMC5148198

[17] Yang H, Xiang Y, Robinson K, Wang J, Zhang G, Zhao J, Xiao Y. 2018. Gut microbiota is a major contributor to adiposity in pigs. Front Microbiol 9:3045. doi:10.3389/fmicb.2018.03045 30619136 PMC6296290

[18] He B, Bai Y, Jiang L, Wang W, Li T, Liu P, Tao S, Zhao J, Han D, Wang J. 2018. Effects of oat bran on nutrient digestibility, intestinal microbiota, and inflammatory responses in the hindgut of growing pigs. Int J Mol Sci 19:2407. doi:10.3390/ijms19082407 30111703 PMC6121460

[19] Xiao Y, Kong F, Xiang Y, Zhou W, Wang J, Yang H, Zhang G, Zhao J. 2018. Comparative biogeography of the gut microbiome between Jinhua and Landrace pigs. Sci Rep 8:5985. doi:10.1038/s41598-018-24289-z 29654314 PMC5899086

[20] Ma L, Lyu W, Song Y, Chen K, Lv L, Yang H, Wang W, Xiao Y. 2023. Anti-inflammatory effect of Clostridium butyricum-derived extracellular vesicles in ulcerative colitis: impact on host microRNAs expressions and gut microbiome profiles. Molecular Nutrition Food Res 67:e2200884. doi:10.1002/mnfr.202370029 37183784

[21] Wang X, Tsai T, Deng F, Wei X, Chai J, Knapp J, Apple J, Maxwell CV, Lee JA, Li Y, Zhao J. 2019. Longitudinal investigation of the swine gut microbiome from birth to market reveals stage and growth performance associated bacteria. Microbiome 7:109. doi:10.1186/s40168-019-0721-7 31362781 PMC6664762

[22] Pabst R. 2020. The pig as a model for immunology research. Cell Tissue Res 380:287–304. doi:10.1007/s00441-020-03206-9 32356014 PMC7223737

[23] Xiao L, Estellé J, Kiilerich P, Ramayo-Caldas Y, Xia Z, Feng Q, Liang S, Pedersen AØ, Kjeldsen NJ, Liu C, Maguin E, Doré J, Pons N, Le Chatelier E, Prifti E, Li J, Jia H, Liu X, Xu X, Ehrlich SD, Madsen L, Kristiansen K, Rogel-Gaillard C, Wang J. 2016. A reference gene catalogue of the pig gut microbiome. Nat Microbiol 1:16161. doi:10.1038/nmicrobiol.2016.161 27643971

[24] Shenhav L, Thompson M, Joseph TA, Briscoe L, Furman O, Bogumil D, Mizrahi I, Pe’er I, Halperin E. 2019. FEAST: fast expectation-maximization for microbial source tracking. Nat Methods 16:627–632. doi:10.1038/s41592-019-0431-x 31182859 PMC8535041

[25] Yuan C, Zhang P, Liu P, Li Y, Li J, Zhang E, Jin Y, Yang Q. 2022. A novel pathway for porcine epidemic diarrhea virus transmission from sows to neonatal piglets mediated by colostrum. J Virol 96:e0047722. doi:10.1128/jvi.00477-22 35758666 PMC9327711

[26] Grześkowiak ŁM, Pieper R, Huynh HA, Cutting SM, Vahjen W, Zentek J. 2019. Impact of early-life events on the susceptibility to Clostridium difficile colonisation and infection in the offspring of the pig. Gut Microbes 10:251–259. doi:10.1080/19490976.2018.1518554 30252612 PMC6546313

[27] Li J, Chen D, Yu B, He J, Huang Z, Mao X, Zheng P, Yu J, Luo J, Tian G, Luo Y. 2020. The fungal community and its interaction with the concentration of short-chain fatty acids in the faeces of Chenghua, Yorkshire and Tibetan pigs. Microb Biotechnol 13:509–521. doi:10.1111/1751-7915.13507 31691493 PMC7017814

[28] Davis EC, Wang M, Donovan SM. 2022. Microbial interrelationships across sites of breastfeeding mothers and infants at 6 weeks postpartum. Microorganisms 10:1155. doi:10.3390/microorganisms10061155 35744673 PMC9230604

[29] Scupham AJ, Presley LL, Wei B, Bent E, Griffith N, McPherson M, Zhu F, Oluwadara O, Rao N, Braun J, Borneman J. 2006. Abundant and diverse fungal microbiota in the murine intestine. Appl Environ Microbiol 72:793–801. doi:10.1128/AEM.72.1.793-801.2006 16391120 PMC1352209

[30] Hallen-Adams HE, Kachman SD, Kim J, Legge RM, Martínez I. 2015. Fungi inhabiting the healthy human gastrointestinal tract: a diverse and dynamic community. Fungal Ecol 15:9–17. doi:10.1016/j.funeco.2015.01.006

[31] Kong Q, Liu S, Li A, Wang Y, Zhang L, Iqbal M, Jamil T, Shang Z, Suo LS, Li J. 2021. Characterization of fungal microbial diversity in healthy and diarrheal Tibetan piglets. BMC Microbiol 21:204. doi:10.1186/s12866-021-02242-x 34217216 PMC8254304

[32] Richardson MJ. 2001. Diversity and occurrence of coprophilous fungi. Mycol Res 105:387–402. doi:10.1017/S0953756201003884

[33] Kittelmann S, Naylor GE, Koolaard JP, Janssen PH. 2012. A proposed taxonomy of anaerobic fungi (class Neocallimastigomycetes) suitable for large-scale sequence-based community structure analysis. PLoS One 7:e36866. doi:10.1371/journal.pone.0036866 22615827 PMC3353986

[34] Prisnee TL, Fouhse JM, Diether NE, Lantz HL, Ju T, Willing BP. 2022. Maternal mycobiome, but not antibiotics, alters fungal community structure in neonatal piglets. Appl Environ Microbiol 88:e0159322. doi:10.1128/aem.01593-22 36448784 PMC9765005

[35] Hu J, Chen J, Hou Q, Xu X, Ren J, Ma L, Yan X. 2023. Core-predominant gut fungus Kazachstania slooffiae promotes intestinal epithelial glycolysis via lysine desuccinylation in pigs. Microbiome 11:31. doi:10.1186/s40168-023-01468-3 36814349 PMC9948344

[36] Zhou X, He Y, Chen J, Xiong X, Yin J, Liang J, Peng C, Huang C, Guan G, Yin Y. 2023. Colonic phosphocholine is correlated with Candida tropicalis and promotes diarrhea and pathogen clearance. NPJ Biofilms Microbiomes 9:62. doi:10.1038/s41522-023-00433-0 37666845 PMC10477305

[37] Borruso L, Checcucci A, Torti V, Correa F, Sandri C, Luise D, Cavani L, Modesto M, Spiezio C, Mimmo T, Cesco S, Di Vito M, Bugli F, Randrianarison RM, Gamba M, Rarojoson NJ, Zaborra CA, Mattarelli P, Trevisi P, Giacoma C. 2021. I like the way you eat it: lemur (Indri indri) gut mycobiome and geophagy. Microb Ecol 82:215–223. doi:10.1007/s00248-020-01677-5 33471174 PMC8282574

[38] Bani A, Borruso L, Matthews Nicholass KJ, Bardelli T, Polo A, Pioli S, Gómez-Brandón M, Insam H, Dumbrell AJ, Brusetti L. 2019. Site-specific microbial decomposer communities do not imply faster decomposition: results from a litter transplantation experiment. Microorganisms 7:349. doi:10.3390/microorganisms7090349 31547404 PMC6780308

[39] Eustis SL, Kirkbride CA, Gates C, Haley LD. 1981. Porcine abortions associated with fungi, Actinomycetes, and Rhodococcus sp. Vet Pathol 18:608–613. doi:10.1177/030098588101800505 7281459

[40] Li Z, Lu G, Meng G. 2019. Pathogenic fungal infection in the lung. Front Immunol 10:1524. doi:10.3389/fimmu.2019.01524 31333658 PMC6616198

[41] Sabino R, Faísca VM, Carolino E, Veríssimo C, Viegas C. 2012. Occupational exposure to Aspergillus by swine and poultry farm workers in Portugal. J Toxicol Environ Health Part A 75:1381–1391. doi:10.1080/15287394.2012.721170 23095156

[42] Todd JN, Wells GA, Davie J. 1985. Mycotic abortion in the pig. Vet Rec 116:350. doi:10.1136/vr.116.13.350 4002544

[43] Briard B, Mislin GLA, Latgé J-P, Beauvais A. 2019. Interactions between Aspergillus fumigatus and pulmonary bacteria: current state of the field, new data, and future perspective. J Fungi (Basel) 5:48. doi:10.3390/jof5020048 31212791 PMC6617096

[44] Melloul E, Roisin L, Durieux MF, Woerther PL, Jenot D, Risco V, Guillot J, Dannaoui E, Decousser JW, Botterel F. 2018. Interactions of Aspergillus fumigatus and Stenotrophomonas maltophilia in an in vitro mixed biofilm model: does the strain matter? Front Microbiol 9. doi:10.3389/fmicb.2018.02850 PMC6277776 30542331

[45] Schroeckh V, Scherlach K, Nützmann H-W, Shelest E, Schmidt-Heck W, Schuemann J, Martin K, Hertweck C, Brakhage AA. 2009. Intimate bacterial-fungal interaction triggers biosynthesis of archetypal polyketides in Aspergillus nidulans. Proc Natl Acad Sci USA 106:14558–14563. doi:10.1073/pnas.0901870106 19666480 PMC2732885

[46] Mucci MJ, Cuestas ML, Landanburu MF, Mujica MT. 2017. Prevalence of Candida albicans, Candida dubliniensis and Candida africana in pregnant women suffering from vulvovaginal candidiasis in Argentina. Rev Iberoam Micol 34:72–76. doi:10.1016/j.riam.2016.09.001 28385421

[47] Shi XY, Yang YP, Zhang Y, Li W, Wang JD, Huang WM, Fan YM. 2015. Molecular identification and antifungal susceptibility of 186 Candida isolates from vulvovaginal candidiasis in southern China. J Med Microbiol 64:390–393. doi:10.1099/jmm.0.000024 25596116

[48] Yang T, Tedersoo L, Soltis PS, Soltis DE, Gilbert JA, Sun M, Shi Y, Wang H, Li Y, Zhang J, Chen Z, Lin H, Zhao Y, Fu C, Chu H. 2019. Phylogenetic imprint of woody plants on the soil mycobiome in natural mountain forests of eastern China. ISME J 13:686–697. doi:10.1038/s41396-018-0303-x 30353037 PMC6461945

[49] Averill C, Hawkes CV. 2016. Ectomycorrhizal fungi slow soil carbon cycling. Ecol Lett 19:937–947. doi:10.1111/ele.12631 27335203

[50] Velie BD, Maltecca C, Cassady JP. 2009. Genetic relationships among pig behavior, growth, backfat, and loin muscle area. J Anim Sci 87:2767–2773. doi:10.2527/jas.2008-1328 19542514

[51] Schoch CL, Seifert KA, Huhndorf S, Robert V, Spouge JL, Levesque CA, Chen W, Fungal Barcoding Consortium, Fungal Barcoding Consortium Author List. 2012. Nuclear ribosomal internal transcribed spacer (ITS) region as a universal DNA barcode marker for Fungi. Proc Natl Acad Sci USA 109:6241–6246. doi:10.1073/pnas.1117018109 22454494 PMC3341068

[52] Gardes M, Bruns TD. 1993. ITS primers with enhanced specificity for basidiomycetes - application to the identification of mycorrhizae and rusts. Mol Ecol 2:113–118. doi:10.1111/j.1365-294x.1993.tb00005.x 8180733

[53] Bolyen E, Rideout JR, Dillon MR, Bokulich NA, Abnet CC, Al-Ghalith GA, Alexander H, Alm EJ, Arumugam M, Asnicar F, et al.. 2019. Reproducible, interactive, scalable and extensible microbiome data science using QIIME 2. Nat Biotechnol 37:852–857. doi:10.1038/s41587-019-0209-9 31341288 PMC7015180

[54] Callahan BJ, McMurdie PJ, Rosen MJ, Han AW, Johnson AJA, Holmes SP. 2016. DADA2: high-resolution sample inference from Illumina amplicon data. Nat Methods 13:581–583. doi:10.1038/nmeth.3869 27214047 PMC4927377

[55] Katoh K, Misawa K, Kuma K, Miyata T. 2002. MAFFT: a novel method for rapid multiple sequence alignment based on fast Fourier transform. Nucleic Acids Res 30:3059–3066. doi:10.1093/nar/gkf436 12136088 PMC135756

[56] Bokulich NA, Kaehler BD, Rideout JR, Dillon M, Bolyen E, Knight R, Huttley GA, Gregory Caporaso J. 2018. Optimizing taxonomic classification of marker-gene amplicon sequences with QIIME 2’s q2-feature-classifier plugin. Microbiome 6:90. doi:10.1186/s40168-018-0470-z 29773078 PMC5956843

[57] McMurdie PJ, Holmes S. 2013. phyloseq: an R package for reproducible interactive analysis and graphics of microbiome census data. PLoS One 8:e61217. doi:10.1371/journal.pone.0061217 23630581 PMC3632530

[58] Oksanen JAI, Blanchet FG, Kindt R, Legendre P, O’Hara R, Simpson G, Minchin PEH, O’Hara R. vegan: community ecology package. R package version 1.8-5

[59] Team RC. 2014. R: a language and environment for statistical computing. MSOR connections 1

[60] Liu L, Song C, Wu Z, Xu H, Li J, Wang B, Li J. 2023. GPR clutter removal based on weighted nuclear norm minimization for nonparallel cases. Sensors (Basel) 23:5078. doi:10.3390/s23115078 37299806 PMC10255216

[61] Hamilton NE, Ferry MW. 2018. ggtern: ternary diagrams using ggplot2. J Stat Softw 87:1–17. doi:10.18637/jss.v087.c03

[62] Ward DV, Hoss AG, Kolde R, van Aggelen HC, Loving J, Smith SA, Mack DA, Kathirvel R, Halperin JA, Buell DJ, Wong BE, Ashworth JL, Fortunato-Habib MM, Xu L, Barton BA, Lazar P, Carmona JJ, Mathew J, Salgo IS, Gross BD, Ellison RT. 2019. Integration of genomic and clinical data augments surveillance of healthcare-acquired infections. Infect Control Hosp Epidemiol 40:649–655. doi:10.1017/ice.2019.75 31012399

[63] Nilsson RH, Larsson KH, Taylor AFS, Bengtsson-Palme J, Jeppesen TS, Schigel D, Kennedy P, Picard K, Glöckner FO, Tedersoo L, Saar I, Kõljalg U, Abarenkov K. 2019. The UNITE database for molecular identification of fungi: handling dark taxa and parallel taxonomic classifications. Nucleic Acids Res 47:D259–D264. doi:10.1093/nar/gky1022 30371820 PMC6324048

[64] Nguyen NH, Song Z, Bates ST, Branco S, Tedersoo L, Menke J, Schilling JS, Kennedy PG. 2016. FUNGuild: an open annotation tool for parsing fungal community datasets by ecological guild. Fungal Ecol 20:241–248. doi:10.1016/j.funeco.2015.06.006

